# Stereopsis impairment is associated with decreased color perception and worse motor performance in Parkinson’s disease

**DOI:** 10.1186/2047-783X-19-29

**Published:** 2014-05-24

**Authors:** Liang Sun, Hui Zhang, Zhuqin Gu, Ming Cao, Dawei Li, Piu Chan

**Affiliations:** 1 Department of Neurology, Xuanwu Hospital of Capital Medical University, 45 Changchun Street, Beijing 100053, China; 2 Department of Neurobiology, Beijing Institute of Geriatrics, Xuanwu Hospital of Capital Medical University, 45 Changchun Street, Beijing 100053, China; 3 Key Laboratory on Neurodegenerative Disease of Ministry of Education and Key Laboratory on Parkinson’s Disease of Beijing, 45 Changchun Street, Beijing 100053, China

**Keywords:** Color perception, Parkinson’s disease, Stereopsis

## Abstract

**Background:**

We conducted this study is to investigate the correlation between stereopsis dysfunction and color perception, as well as whether stereopsis impairment is associated with motor dysfunction in patients with Parkinson’s disease (PD).

**Method:**

Our present study included 45 PD patients and 50 non-PD control patients attending the Movement Disorder Center at Xuanwu Hospital Capital Medical University in Beijing from July 2011 to November 2011. Neurologic evaluations and visual function assessments were conducted, and the results between two groups of patients were compared.

**Results:**

We found that the total error scores (TESs) and partial error scores (PESs) for red, green, blue and purple were all significantly higher in PD patients than in control patients. The limited grade on the FLY Stereo Acuity Test with LEA Symbols was significantly lower in PD patients than in control patients (*P* = 0.0001), whereas the percentage of abnormal stereopsis in PD patients was significantly higher than in control patients (42.2% vs. 12%; *P* = 0.001). Multiple linear regression analysis showed that PD patients with higher Hoehn and Yahr Scale stage, and those with decreased stereopsis had higher Unified Parkinson’s Disease Rating Scale (UPDRS) motor scores and worse motor function. Furthermore, our study demonstrates that the UPDRS motor scores and total average number of the Purdue Pegboard Test scores of PD patients were significantly improved when they had taken their medications, and the TESs and PESs for green were lower in when they were off their medications.

**Conclusion:**

Our results provide more information on the underlying mechanisms of vision, motor and stereopsis impairments in PD patients.

## Background

Visual dysfunction is a common nonmotor symptom in Parkinson’s disease (PD) that has several manifestations. The impairment in color discrimination is one of the most well-established visual deficits in PD patients [1-3]. It can be caused by peripheral retinal dopaminergic deficiency [4-6] or by central visual impairments such as orientation impairments [7,8], motion detection deficit [9,10] and abnormal visual attention [11,12].

Recently, in a small study of PD patients, researchers found stereopsis dysfunction to be related to visual cognitive dysfunction [13]. Interestingly, to date, there has been no simultaneous assessment of the potential influence of peripheral abnormality on stereopsis impairment, which could also be triggered once normal cortex is fed with erroneous information from peripheral pathways such as retinal ganglion cells.

In our present cross-sectional study, we first examined the putative interdependence of color perception and stereopsis abnormalities between PD patients and age-matched control patients. We then analyzed the correlations between visual deficits and patients’ demographic features or motor dysfunction. Moreover, we compared the visual function of PD patients between the on and off states to try to further clarify the correlation of stereopsis function with color perception, visual function and motor function in PD patients. Because color perception can be improved with levodopa therapy, we tested whether this treatment can also improve stereopsis dysfunction.

## Methods

### Patients

Between July 2011 and November 2011, 114 PD patients were recruited at the Movement Disorder Center at Xuanwu Hospital Capital Medical University in Beijing, China, to participate in the study. All patients signed the consent forms for participation. All experimental and clinical procedures were reviewed and approved by the Ethics Committee of Xuanwu Hospital, Capital Medical University.

The diagnosis of movement disorders in PD patients was made according to the UK Parkinson’s Disease Society Brain Bank clinical diagnostic criteria [14]. Patients with identified brain lesions or any other neurological disorders were excluded. Patients’ spouses and relatives were screened to participate in this study as age-matched controls. All participants underwent tests for visual acuity and strabismus. To eliminate the influence of ametropia, corrective lenses were permitted during the stereopsis and color perception tests. The exclusion criteria were presence of strabismus, nystagmus, ocular motility disturbance or poor visual acuity, even after correction in either eye (<20/40 Snellen fraction). Furthermore, the patients were asked about their history of eye diseases. Those with a history of unoperated symptomatic cataracts, untreated glaucoma, diabetic eye disease, congenital color blindness or significant cognitive impairment were also excluded from the study. After applying these criteria, 19 of the 114 patients were excluded from the study.

### Neurologic evaluation

Standardized neurologic examinations and clinical assessments were performed. All evaluations were carried out in the morning, and patients were asked not to take any antiparkinsonian medications for at least 12 hours (18 hours for combined levodopa and carbidopa) to ensure a practically defined off state [15]. The Hoehn and Yahr (H&Y) stage and Unified Parkinson’s Disease Rating Scale (UPDRS) scores were determined. The Purdue Pegboard Test (PPT) was used to test hand dexterity and motor speed. Briefly, this test requires the person to place as many pins as possible in vertical columns of holes on a board within 30 seconds. The pins were placed in the right, left and both hands three times for each, and the average number of the pins placed under the three conditions were recorded. The total average number (TAN) of Purdue Pegboard Test scores was used as the measurement method.

Seven patients who had obvious “on and off” phenomenon were evaluated on the basis of two sets of UPDRS score, H&Y stage and PPT scores: after they had taken or not taken their usual antiparkinsonian medication. The Mini Mental State Examination (MMSE) and Montreal Cognitive Assessment (MoCA) were used to assess patients’ cognitive function.

### Visual function assessment

Patients were evaluated for visual function in the morning in while not taking medication. All participants underwent tests of visual acuity and strabismus. Their medical histories of eye diseases were also recorded. Patients were excluded from the study if they had strabismus, nystagmus, ocular motility disturbance and/or poor visual acuity, even after correction in either eye (<20/40 Snellen fraction). In addition, patients with any of the following conditions were excluded on the basis of a history of any of the following: unoperated symptomatic cataracts, untreated glaucoma, diabetic eye disease, congenital color blindness and/or significant cognitive impairment. All visual function tests were conducted in natural daylight while avoiding direct sunlight. Stereopsis was assessed using the FLY Stereo Acuity Test with LEA Symbols (P/N 1000; Vision Assessment, Elk Grove Village, IL, USA). This test includes 10 grades, in descending order from 400 to 20 seconds of arc. The limited grade (LG) of the FLY Stereo Acuity Test with LEA Symbols was used as the index of participants’ stereopsis function. *Normal stereopsis* was defined as LG ≥5 (arc ≥63 seconds). Color perception was tested using Farnsworth-Munsell 100-Hue Test scores without time limits. The total error scores (TESs) and partial error scores (PESs) for red (caps 85 to 21), green (caps 22 to 42), blue (caps 43 to 63) and purple (caps 64 to 85) were measured.

### Statistical analysis

Data were analyzed using SPSS version 11.5 software (SPSS, Chicago, IL, USA). As age might influence visual function [16], comparisons of visual function between PD patients and controls were performed with one-way analysis of variance, including age as a confounding covariate. To assess the characteristics of participants with normal or abnormal stereopsis, a *t*-test was carried out. The logistic regression model was used to determine the independent risk factors for stereopsis impairment in PD patients. The multiple linear regression model was used to study the independent factors affecting color perception and UPDRS part III score in PD patients. Visual and motor functions in PD patients while taking or not taking medication were assessed by performing a paired *t*-test. Differences were considered to be significant if the *P*-value was <0.05.

## Results

### Patient characteristics

A total of 114 participants, comprising 53 PD patients and 61 non-PD patients (controls), were included in this study. From among these participants, 19 were excluded because of concomitant brain lesions (*n* = 3), poor visual acuity (*n* = 8), symptomatic cataracts (*n* = 6) or congenital color blindness (*n* = 2). As a result, 45 PD patients (26 men and 19 women, mean age of 65.40 years, range from 45 to 81 years) and 50 controls (22 men and 28 women, mean age of 65.48 years, range from 47 to 83 years) were enrolled into the study. The PD patients had the following characteristics: mean H&Y stage 2.22 ± 0.68, mean UPDRS motor score 25.18 ± 13.06, mean MMSE score 27.50 ± 3.09, mean MoCA score 23.95 ± 5.03 and mean disease duration 6.60 ± 4.52 years. The mean TAN (PPT scores) in PD patients was 34.28 ± 10.12, which is significantly lower than the mean score of 49.56 ± 6.50 in controls (*P* = 0.0001). TAN (PPT scores) was correlated with UPDRS total score, UPDRS motor score and H&Y stage in PD patients (*P* = 0.0001, *P* = 0.0001 and *P* = 0.029, respectively). However, they were not associated with MMSE score, MoCA score or disease duration (*P* = 0.519, *P* = 0.135 and *P* = 0.160, respectively). TAN (PPT scores) was correlated with age in controls (*P* = 0.0001), as younger control participants had better values than elderly ones. Overall, the demographic features of participants and the clinical rating scales used to assess PD patients are listed in Table 1. The TAN correlation factors are shown in Table 2.

**Table 1:** **Demographic features of the patients**^**a**^

**Demographic features**	**PD patients (*n*** **= 45)**	**Controls (*n*** **= 50)**
Age (yr) (means ± SD)	65.40 ± 9.37	65.48 ± 7.97
Sex (% male)	57.8	44.0
Body mass index (kg/m^2^) (means)	29.2	28.6
Marital status (ever-married) (n)	40	42
Duration of education (yr) (means)	8.7	7.9
Annual household income (Chinese yuan) (means)	64,320	62,325
H&Y stage (means ± SD)	2.22 ± 0.68	NA
UPDRS part III score (means ± SD)	25.18 ± 13.06	NA
UPDRS total score (means ± SD)	42.47 ± 19.71	NA
TAN (PPT scores) (means ± SD)	34.28 ± 10.12^b^	49.56 ± 6.50
MMSE score (means ± SD)	27.50 ± 3.09	28.36 ± 2.44
MoCA score (means ± SD)	23.95 ± 5.03	24.64 ± 4.37
Motor disease duration (yr) (means ± SD)	6.60 ± 4.52	NA
Hypertension (*n*)	8	10
Hyperthyroidism (*n*)	2	3
Diabetes (*n*)	9	8

^a^H&Y, Hoehn and Yahr; MMSE, Mini Mental State Examination; MoCA, Montreal Cognitive Assessment; NA, Not applicable; PD, Parkinson’s disease; PPT, Purdue Pegboard Test; TAN, Total average number of Purdue Pegboard Test scores; UPDRS, Unified Parkinson’s Disease Rating Scale. ^b^*P* = 0.0001.

**Table 2:** **Pearson correlations between total average number of Purdue Pegboard Test scores and other factors**^**a**^

**Data parameters**	**PD patients (*n*** **= 45)**		**Controls (*n*** **= 50)**	
	**Correlation coefficient (*t*)**	***P***	**Correlation coefficient (*t*)**	***P***
Age (yr)	-0.127	0.405	-0.633	0.0001^c^
Sex (% male)	-0.196	0.845	-1.715	0.093
H&Y stage	-0.326	0.029^b^	NA	NA
UPDRS part III score	-0.620	0.0001^c^	NA	NA
UPDRS total score	-0.579	0.0001^c^	NA	NA
MMSE score	0.100	0.519	0.070	0.758
MoCA score	0.237	0.135	0.312	0.158
Course of disease	-0.229	0.160	NA	NA

^a^H&Y, Hoehn and Yahr stage; MMSE, Mini Mental State Examination; MoCA, Montreal Cognitive Assessment; PD, Parkinson’s disease; UPDRS, Unified Parkinson’s Disease Rating Scale. ^b^*P* < 0.05, ^c^*P* < 0.01.

### Visual function comparison between Parkinson’s disease patients and controls

We compared stereopsis and color perception between PD patients and controls after matching for age between the two groups. For color perception, the TES and PES for red, green, blue and purple were all significantly higher in PD patients than in controls (*P* = 0.0001, *P* = 0.001, *P* = 0.0001, *P* = 0.001 and *P* = 0.0001, respectively). The LG values on the FLY Stereo Acuity Test with LEA Symbols were significantly lower in PD patients than in controls (*P* = 0.0001), and the percentage of abnormal stereopsis in PD patients (42.2%) was significantly higher than that in controls (12.0%) (*P* = 0.001). The comparisons of color perception and stereopsis are presented in Table 3.

**Table 3:** **Comparison of visual function in Parkinson’s disease patients and controls (adjusted for age)**^**a**^

**Visual function measurements**	**PD patients (*n*** **= 45)**	**Controls(*n*** **= 50)**	**Adjusted** ***P*-value**
Color perception (Farnsworth-Munsell 100-Hue Test score)			
TES **(mean ± SD)**	135.82 ± 84.78	84.26 ± 41.73	0.0001^b^
PES (red) **(mean ± SD)**	33.18 ± 26.65	20.14 ± 14.40	0.001^c^
PES (green) **(mean ± SD)**	31.04 ± 17.66	19.90 ± 13.00	0.0001^b^
PES (blue) **(mean ± SD)**	45.33 ± 30.71	29.74 ± 14.35	0.001^c^
PES (purple) **(mean ± SD)**	26.22 ± 21.74	14.48 ± 10.35	0.0001^b^
Stereopsis			
LG **(mean ± SD)**	5.16 ± 2.59	7.10 ± 1.89	0.0001^b^
Abnormal (%)	42.2%	12.0%	0.001^c^

^a^LG, Limited grade; PD, Parkinson’s disease; PES, Partial error score; TES, Total error score. ^b^P < 0.001; ^c^P < 0.05.

### Factors influencing color perception in Parkinson’s disease patients

A multiple linear regression model was used to study the factors influencing color perception in PD patients, with TES used as the dependent variable. The probed stereopsis factors included age, H&Y stage, UPDRS total score, duration of disease, MMSE score, MoCA score, TAN (PPT scores) and LG values. Our results show that only age was independently and significantly associated with TES in PD patients (*t* = 2.583, *P* = 0.015).

### Factors associated with stereopsis function in Parkinson’s disease patients

To study the factors associated with stereopsis function, PD patients with normal stereopsis (PDNS) and PD patients with abnormal stereopsis (PDAS) were compared. These data are shown in Table 4. We found no significant differences with regard to age, H&Y stage, disease duration or MMSE and MoCA scores between PDNS and PDAS. However, in PDAS, UPDRS total scores and UPDRS motor scores were higher (*P* = 0.033 and *P* = 0.018, respectively), whereas TAN (PPT scores) was lower (*P* = 0.014). The TES and the PES for green and purple were significantly higher in PDAS than in PDNS (*P* = 0.019, *P* = 0.001 and *P* = 0.039, respectively). To study the independent risk factors for stereopsis impairment in PD patients, we used a logistic regression model. The factors with significant mean values in one-factor analysis were examined using this model. The results show that only the PES for green remained significant, as the adjusted *P*-value was 0.027 and the odds ratio (OR) of the PES for green was 1.093 (approximately 1.010 to 1.182). In addition, when we compared normal and abnormal stereopsis in the controls, we found no significant differences in TAN (PPT scores), TES or PESs in FMT(Farnsworth-Munsell 100-Hue Test).

**Table 4:** **Comparison of Parkinson’s disease patients regarding normal vs abnormal stereopsis**^**a**^

**Data parameters**	**PD patients (*N*** **= 45)**			
	**Normal stereopsis (*n*** **= 26) (mean ± SD)**	**Abnormal stereopsis (*n*** **= 19) (mean ± SD)**	***P*-value**	**Adjusted** ***P*-value**
Age (yr)	64.58 ± 8.31	66.53 ± 10.79	0.497	
H&Y stage	2.04 ± 0.73	2.40 ± 0.59	0.089	
UPDRS part III score	20.81 ± 7.48	31.16 ± 16.54	0.018^b^	0.112
UPDRS total score	37.15 ± 13.96	49.74 ± 24.14	0.033^b^	
TAN (PPT scores)	37.41 ± 9.48	30.00 ± 9.61	0.014^b^	0.575
MMSE score	28.04 ± 1.74	26.78 ± 4.22	0.187	
MoCA score	24.96 ± 3.70	22.67 ± 6.22	0.150	
Line connection	0.66 ± 0.48	0.61 ± 0.50	0.692	
Copy cube	0.76 ± 0.44	0.55 ± 0.51	0.083	
Drawing clock	2.31 ± 0.97	2.06 ± 1.06	0.311	
Disease duration (yr)	6.59 ± 5.34	6.61 ± 3.34	0.989	
TES	110.85 ± 65.19	170.00 ± 97.63	0.019^b^	
PES (red)	27.23 ± 23.31	41.32 ± 29.33	0.08	
PES (green)	23.96 ± 14.61	40.74 ± 17.17	0.001^c^	0.027^b^
PES (blue)	39.04 ± 25.08	53.95 ± 36.00	0.108	
PES (purple)	20.54 ± 14.78	34.00 ± 27.23	0.039^b^	0.409

^a^H&Y, Hoehn and Yahr; MMSE, Mini Mental State Examination; MoCA, Montreal Cognitive Assessment; PD, Parkinson’s disease; PES, Partial error score; PPT, Purdue Pegboard Test; TAN, total average number; TES, Total error score; UPDRS, Unified Parkinson’s Disease Rating Scale. ^*b*^P < 0.05; ^c^*P* < 0.01. For the adjusted *P*-value, a logistic regression model was used with stereopsis (normal or abnormal) as the dependent variable.

The influence of visual function on motor symptoms in PD patients was analyzed using a multiple linear regression model. The UPDRS motor score was considered the dependent variable, and age, H&Y stage, course of disease, MMSE and MoCA scores, TES and LG values on the FLY Stereo Acuity Test with LEA Symbols were used as independent variables. Our results show that LG values on the FLY Stereo Acuity Test with LEA Symbols (*P* = 0.008) and H&Y stage (*P* = 0.018) were independently associated with UPDRS motor scores. Our results also show that PD patients with higher H&Y stages and decreased stereopsis had higher UPDRS motor scores and worse motor function. PDAS had higher scores on the UPDRS part III in rigidity, finger taps, hand movements, rapid alternating movements and leg agility, than those of PDNS (*P* = 0.009, *P* = 0.001, *P* = 0.036, *P* = 0.007 and *P* = 0.007, respectively) (Table 5).

**Table 5:** **Comparison of Unified Parkinson’s Disease Rating Scale motor scores between normal and abnormal stereopsis in Parkinson’s disease patients**^**a**^

**Patient characteristics**	**PDNS (n = 26) (mean ± SD)**	**PDAS (n = 19) (mean ± SD)**	***P*-value**
Speech	0.42 ± 0.64	0.84 ± 1.02	0.097
Facial expression	1.04 ± 0.77	1.42 ± 0.61	0.081
Tremor at rest	2.15 ± 2.84	4.05 ± 3.81	0.063
Action or postural tremor of hands	0.85 ± 0.88	1.32 ± 1.25	0.146
Rigidity	4.88 ± 2.85	7.47 ± 3.50	0.009^b^
Finger taps	1.46 ± 1.07	2.79 ± 1.48	0.001^b^
Hand movements	1.73 ± 1.04	2.42 ± 1.07	0.036^c^
Rapid alternating movements	1.38 ± 1.06	2.47 ± 1.50	0.007^b^
Leg agility	1.12 ± 1.31	2.21 ± 1.27	0.007^b^
Arising from chair	0.69 ± 1.49	0.58 ± 1.07	0.779
Posture	1.08 ± 0.56	1.11 ± 0.57	0.868
Gait	1.08 ± 0.65	0.95 ± 0.62	0.468
Posture stability	1.04 ± 1.22	0.84 ± 0.60	0.521
Body bradykinesia and hypokinesia	1.35 ± 0.80	1.63 ± 0.83	0.250

^a^PDAS, Parkinson’s disease patients with abnormal stereopsis; PDNS, Parkinson’s disease patients with normal stereopsis. ^b^*P* < 0.01; ^c^*P* < 0.05.

### Comparison of visual function between medication on and off states in Parkinson’s disease patients

The UPDRS motor scores and TAN (PPT scores) of the 16 PD patients were significantly improved while taking medications (*P* < 0.05 and *P* < 0.05, respectively) (Table 6). TES and PESs for green were lower while taking medications vs not (*P* < 0.05). We found no difference in stereopsis between when the patients were or were not taking medication (*P* > 0.05).

**Table 6:** **Paired comparison of motor function and visual function in 16 Parkinson’s disease patients taking vs not taking medications**^**a**^

**Data parameters**	**Off medication (*n*** **= 8) (mean ± SD)**	**On medication (*n*** **= 9) (mean ± SD)**	***t***	***P*-value**
H&Y stage	2.11 ± 0.50	1.94 ± 0.52	1.902	0.110
UPDRS part III score	27.37 ± 13.01	10.24 ± 3.51	3.680	0.009^b^
TAN (PPT scores)	35.74 ± 8.65	44.33 ± 6.57	-3.78	0.007^b^
TES	89.24 ± 30.74	60.43 ± 30.55	2.764	0.025^c^
PES (red)	12.43 ± 6.53	9.87 ± 7.95	1.114	0.376
PES (green)	23.14 ± 9.34	12.56 ± 8.12	2.545	0.027^c^
PES (blue)	35.49 ± 18.12	29.01 ± 17.50	1.227	0.290
PES (purple)	12.45 ± 7.88	10.67 ± 8.54	0.711	0.574
Stereopsis (LG)	6.33 ± 1.82	6.64 ± 2.07	-1.023	0.377

^a^H&Y, Hoehn and Yahr; LG, Limited grade; MMSE, Mini Mental State Examination; MoCA, Montreal Cognitive Assessment; PES, Partial error score; PPT, Purdue Pegboard Test; TAN, Total average number; TES, Total error score; UPDRS, Unified Parkinson’s Disease Rating Scale. ^b^*P* < 0.05; ^c^*P* < 0.01.

## Discussion

The complex process of stereopsis is governed mainly by the cerebral extrastriatal cortex [17,18]. Stereopsis impairments have been reported in patients with supratentorial lesions [19]. Little is known, however, about the underlying mechanisms of stereopsis dysfunction in Parkinson’s disease, because not only central but also peripheral visual pathways are involved.

In the present study, we show that PD patients made more errors in color discrimination tests and had prominent deficits on the green and purple axes. These results are in line with those reported in previous studies showing that blue cone deficiency is predominant in older PD patients [20] and that nonmotor impairments, including color discrimination deficits, are highly associated with PD [2,4]. Interestingly, our study demonstrates that color discrimination deficits were independent of the progression of PD, suggesting that the factor of age might be the sole independent risk factor to consider in determining the severity of retinal impairment in PD patients [16].

Our results also demonstrate that more PD patients (19 (42.2%) of 45) than control patients (6 (12%) of 50) had abnormal stereopsis. This result cannot be explained as simply an age-related decrease in stereopsis, as shown in previous studies [21,22], because PD patients and controls were age-matched in our study. Furthermore, we found that PDAS performed significantly worse in motor function tests than PDNS and that abnormal stereopsis was highly associated with decreased color perception in PD patients. Taken together, these results strongly suggest that stereopsis impairment might be closely associated with the progression of PD.

## Conclusions

Overall, the results of our study show that stereopsis impairment is highly associated with color perception and motor dysfunction in PD patients. Multiple factors, in the central as well as peripheral visual pathways, may work congruently in contributing to the pathology of stereopsis. Also, stereopsis impairment severely aggravates motor dysfunction in patients with PD. Thus, our results provide further information on the underlying mechanisms of stereopsis impairment, as well as their correlation with the development of cognitive dysfunction in PD patients.

## Competing interests

The authors declare that they have no competing interests.

## Authors’ contributions

LS was responsible for the study design and manuscript preparation. ZG, MC and DL collected and analyzed the data. PC was the Principal Investigator of the study and was responsible for the study design and manuscript finalization. All authors read and approved the final manuscript.
